# Integration of Ligand-Based Drug Screening with Structure-Based Drug Screening by Combining Maximum Volume Overlapping Score with Ligand Doscking 

**DOI:** 10.3390/ph5121332

**Published:** 2012-12-04

**Authors:** Yoshifumi Fukunishi, Haruki Nakamura

**Affiliations:** 1 Biological Information Research Center (BIRC), National Institute of Advanced Industrial Science and Technology (AIST), 2-3-26, Aomi, Koto-ku, Tokyo 135-0064, Japan; 2 Institute for Protein Research, Osaka University, 3-2 Yamadaoka, Suita, Osaka 565-0871, Japan; E-Mail: harukin@protein.osaka-u.ac.jp (H.N.)

**Keywords:** virtual drug screening, structure-based drug screening, protein-compound docking.

## Abstract

Ligand-based and structure-based drug screening methods were integrated for *in silico* drug development by combining the maximum-volume overlap (MVO) method with a protein-compound docking program. The MVO method is used to select reliable docking poses by calculating volume overlaps between the docking pose in question and the known ligand docking pose, if at least a single protein-ligand complex structure is known. In the present study, the compounds in a database were docked onto a target protein that had a known protein-ligand complex structure. The new score is the summation of the docking score and the MVO score, which is the measure of the volume overlap between the docking poses of the compound in question and the known ligand. The compounds were sorted according to the new score. The *in silico* screening results were improved by comparing the MVO score to the original docking score only. The present method was also applied to some target proteins with known ligands, and the results demonstrated that it worked well.

## 1. Introduction

There have been two approaches to *in silico* drug screening: structure-based drug screening (SBDS) and ligand-based drug screening (LBDS). Their key technologies are, respectively, a protein-compound docking program based on the structure of the receptor protein and a molecular similarity calculation program for various chemical compounds.

There have been many reports on LBDS [1,2,3,4,5], which is essentially a similarity search based on known active compounds. The outline of similarity searches is summarized in a review article [5]. To perform a similarity search, one approach is to use a molecular descriptor (a set of many substructures) such as the MACCS key, developed by Molecular Design Limited (MDL; San Leandro, CA, USA) together with a Daylight descriptor (Daylight Chemical Information Systems, Aliso Viejo, CA, USA). Another approach is to overlap the known active compound with compounds selected from a compound library such as ROCS of OpenEye.

Previously, we developed the maximum-volume overlap (MVO) method to improve the docking pose of a compound by using a known protein-ligand complex structure [6]. The MVO method was applied to a molecular dynamics (MD)-MVO method for ligand-based *in silico* drug screening [7]. MD-MVO is a molecular dynamics simulation method for molecular overlapping (alignment). The molecular system consists of a query compound and one or more other compounds selected from a compound library. The intermolecular interaction between compounds is proportional to the molecular overlap instead of the van der Waals and Coulombic interactions between atoms of different molecules. This method was able to achieve both conformer generation of molecules and molecular overlapping (alignment) at the same time. After energy minimization and short-time MD simulation, the molecules in the system were overlapped with each other, and the similarity between compounds was measured according to the volume of the overlap. 

Typically, LBDS and SBDS are used independently. LBDS uses only the known active compounds and not the target protein structure. Conversely, SBDS uses the target protein structure but not the known active compounds [8,9,10,11,12,13,14,15,16,17,18,19,20,21,22], although there are exceptions. The machine-learning multiple target screening (MS-MTS) method uses both the target protein structure and the active compounds [23]. The MS-MTS method requires at least several known active compounds to obtain good screening results, since the MS-MTS method is a sort of machine learning approach. In the MS-MTS method, the docking score is modified by the linear combination of the docking scores with many proteins, as follows:

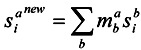
(1)
where S^a^_i_^new^ is the docking score of the i-th compound with the a-th protein and m^a^_b_ is a set of parameters. The parameter m^a^_b_ is optimized to the maximum area under the database enrichment curve (AUC) of known active compounds by a Monte Carlo simulation.

In the present study, we integrated the concepts of LBDS with those of SBDS. Namely, the docking simulation method and the MVO scoring method were combined to introduce the protein force field into the MD-MVO similarity search method. This method requires only a single protein-ligand complex structure instead of multiple ligands as required by the MS-MTS method.

## 2. Results and Discussion

### 2.1. Theoretical Background

**Figure 1:**
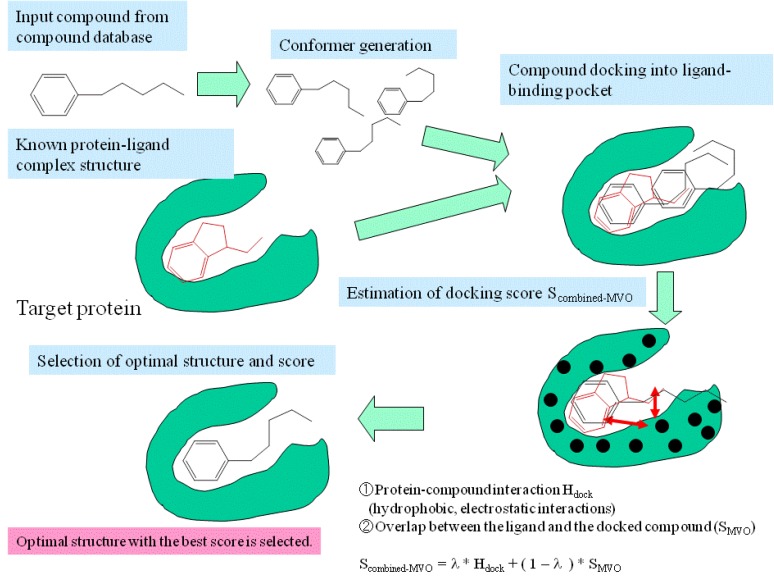
Schematic representation of the combined MVO with docking method.

Figure 1 shows a schematic representation of the present method. To apply this method, one or more protein-ligand complex structures are necessary. The protein-ligand complex structures were obtained by X-ray crystallography, NMR experiments, or computer simulations.

After the compounds in the database were docked onto the target protein, the protein-compound interaction energy and the volume overlap were assessed between the docked compound and the reference ligand that was given *a priori*. The docking score, *S_combined-MVO_*, is the summation of the conventional docking score (*H_dock_*), which is the protein-compound interaction energy, and the MVO score (*S_MVO_*), which is the volume overlap between the docked compound and the reference ligand:

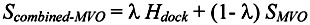
(2)
where λ is a coupling parameter (0 < λ < 1).

The MVO score (*S_MVO_*) is a measure of the volume overlap among the docked compound and the known ligands of the protein-ligand complex structures. We proposed *S_MVO_* in previous papers [6,7]. Let molecules A and B be the template molecule (the known active compound) and the query molecule(s) (the compound in the database), respectively. Let *H_αα_* and *H_αβ_* (=*H_βα_*) be the Hamiltonian of molecule α and the interaction energy between molecules α and β, respectively. Here, α and β are A and B. The total Hamiltonian (*H*) of this system is:

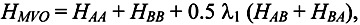
(3)
where λ_1_ is a weight parameter. Here, *H_AA_* and *H_BB_* are the conventional Hamiltonians representing the usual potential energy of atomic interactions within each molecule A and B, respectively.

*H_AB_* (=*H_BA_*) is the newly introduced interaction potential. Let {*x_A_^i^, y_A_^i^, z_A_^i^*} be the {*x, y, z*} coordinates of the i-th atom of molecule A, and let {*x_B_^j^, y_B_^j^, z_B_^j^*} be the {*x, y, z*} coordinates of the j-th atom of molecule B. Let *q_i_^A^* and *q_j_^B^* be the atomic charge of the i-th atom of molecule A and the atomic charge of the j-th atom of molecule B. Then:

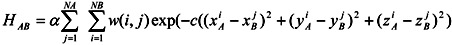
(4)
and:

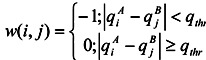
(5)
where α, *N_A_*, *N_B_*, *c*, *w*, and q_thr_ are the conversion factor, the number of atoms in molecule A, the number of atoms in molecule B, a coefficient, a switching function, and a threshold value, respectively. In the present study, parameters α, *c*,and *q_thr_* were set as 1 kcal/mol, 1.0 Å^-2^, and 0.2 in the atomic unit, respectively, as in our previous study [7]. The predicted docking poses are generally 2-3 Å different from the true docking poses, and the errors of the coordinates are larger than the N-H bond lengths. In the amine group, the N and H atoms have negative and positive charges, respectively. Thus the atomic charges of both N and H atoms of amine are set to +0.5 to reduce the error of *H_MVO_*. Also, the atomic charges of all O atoms are set to -0.5 to increase the chance they will overlap. The overlap of O atoms is important in order to maintain the pharmacophore, but the atomic charge of the O atom depends on its neighboring atoms. 

The intramolecular interactions of molecules A and B are calculated by the usual Hamiltonians. These Hamiltonians consist of bond, angle, torsion angle, improper torsion angle, 1-4 and 1-5 van der Waals interactions, and Coulombic interactions. The interaction between A and B is represented by the MVO potential by *H_AB_*. This implies that there are no van der Waals or Coulombic interactions between A and B. 

The score S_AB_ is the measure used to evaluate the overlap between molecules A and B:

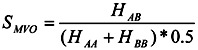
(6)
or:

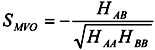
(7)

While many kinds of definitions are available, our screening tests showed that the *S_AB_* by Equation. 6 is the most useful. After the rough docking using the Sievgene docking program [13], the docking poses are optimized based on *S_combined-MVO_* score with Equations. (2) and (6). Test molecules are sorted according to the *S_combined-MVO_* score.

ROCS (OpenEye) calculates the volume overlap between two molecules, while the MVO calculates the volume overlap between two molecules with considering the atomic charges of these molecules. Thus, the MVO method in this study could be replaced by ROCS and the combinations of the other docking programs (Glide, GOLD, DOCK, and etc) and ROCS should be possible.

### 2.2. Examination of Used Parameters and Evaluation of the Combined MVO with Docking Method

The present method was applied to the drug screening of six target proteins: MIF, COX-2, Thermolysin, HIV protease-1, GST, and carboxypeptidase A. Eighteen screening results were obtained. The area under database enrichment curve (AUC) values of the 18 screening results and the hit ratio (% of the active compounds) at the first 1% compounds selected (enrichment factor) are summarized in Table 1 and Table 2. The AUC values are expressed in percentages, and the AUC values of 50% and 100% correspond to the random screening and the ideal screening. The screening results depended on the decoy set used (compound library), so that two decoy sets were used in this examination. The deviations in the AUC values of the 18 screening results are summarized as σ values in Table 1 and Table 2. 

**Table 1:** AUC values (%) obtained by the combined MVO method for LiganBOX decoy set [24].

Damping factor	1	1	1	1	1	1	0.95	0.9	0.85	0.8	MCS
λ	0.0	0.3	0.5	0.7	0.8	1.0	0.5	0.5	0.5	0.5	
18gs	90.2	90.3	90.4	90.4	92.7	67.7	90.5	94.7	96.2	87.3	72.7
1aid	100.0	100.0	99.8	99.5	99.0	93.5	97.6	99.0	98.8	99.9	32.9
1cbx	100.0	100.0	100.0	100.0	97.0	10.0	100.0	100.0	100.0	100.0	100.0
1cox	75.6	75.5	70.2	75.6	69.5	66.3	83.1	76.4	75.2	69.5	54.6
1cps	97.0	99.0	99.0	95.0	88.0	73.0	98.0	97.0	97.0	99.0	100.0
1gcz	55.6	55.9	60.3	59.5	63.5	67.1	61.0	65.5	65.2	61.3	43.7
1hpx	100.0	100.0	99.9	99.9	99.8	89.2	99.2	100.0	100.0	100.0	62.3
1ivp	100.0	99.9	99.6	99.7	99.7	96.4	99.9	100.0	100.0	99.9	67.5
1pxx	71.5	67.2	69.2	70.6	62.3	65.8	70.5	67.7	71.0	68.9	58.4
1tlp	91.2	90.9	89.8	89.4	89.9	49.7	88.6	90.0	89.1	89.5	53.0
1tmn	84.2	84.5	81.4	80.0	79.4	59.6	84.0	89.0	88.4	90.2	52.5
2gss	91.6	90.2	89.0	87.1	90.3	71.5	91.4	81.3	92.3	90.5	41.8
2tmn	90.8	92.2	92.0	90.6	90.3	36.8	91.4	92.2	90.6	91.5	55.4
3cpa	99.0	99.0	99.0	99.0	97.0	88.0	100.0	100.0	100.0	99.0	100.0
3pgh	70.7	70.5	68.4	69.1	65.6	61.0	54.8	64.3	69.9	65.0	79.5
3pgt	90.4	88.0	89.2	92.6	91.3	81.8	91.1	92.5	86.6	88.9	83.2
4cox	66.7	68.7	63.2	64.9	67.0	62.6	68.6	76.4	79.5	73.5	56.7
6cox	81.5	79.6	78.4	81.7	81.5	41.7	87.9	68.4	77.8	76.2	54.6
Average of AUC	86.5	86.2	85.5	85.8	84.7	65.7	86.5	86.3	87.6	86.1	64.9
of AUC	13.0	13.3	13.5	12.8	13.1	21.0	13.5	12.9	11.4	13.1	19.8
1% hit ratio	25.09	29.14	32.27	30.04	24.04	27.09	31.30	25.07	26.63	29.50	19.6

**Table 2:** AUC values (%) obtained by the combined MVO method for Coelacanth decoy set (Coelacanth Chemical Corporation).

Damping factor	1	1	1	1	1	1	0.95	0.9	0.85	0.8	MSC
λ	0	0.3	0.5	0.65	0.8	1	0.5	0.5	0.5	0.5	
18gs	68.2	64.5	63.0	59.0	59.9	35.1	72.3	73.8	65.9	67.7	74.7
1aid	85.1	86.5	78.1	77.4	75.2	69.4	74.2	71.0	77.8	77.9	45.3
1cbx	100.0	100.0	100.0	100.0	98.0	2.0	100.0	100.0	100.0	100.0	100.0
1cox	29.2	37.7	43.4	62.0	64.3	52.5	48.4	40.3	47.4	12.5	67.3
1cps	98.0	99.0	99.0	90.0	72.0	31.0	99.0	96.0	97.0	100.0	100.0
1gcz	27.3	30.9	36.4	43.9	56.1	49.4	36.0	38.6	40.3	31.1	63.3
1hpx	94.0	94.9	87.4	90.0	88.5	56.4	85.1	88.1	84.6	91.4	58.6
1ivp	88.7	88.7	84.5	83.4	81.6	70.7	87.7	88.4	81.8	84.1	63.0
1pxx	21.5	25.3	32.6	43.0	44.7	47.8	22.0	16.4	36.2	27.9	76.1
1tlp	86.5	85.4	85.2	82.0	72.7	29.0	87.4	89.0	82.2	87.8	63.7
1tmn	66.6	66.2	61.0	47.4	40.1	29.4	83.0	87.1	59.2	83.2	55.3
2gss	67.8	69.0	68.0	61.9	54.7	44.8	71.8	68.4	67.5	62.0	68.2
2tmn	87.4	86.5	86.7	83.3	78.6	21.8	88.8	88.7	87.6	88.9	81.7
3cpa	100.0	100.0	100.0	100.0	99.0	83.0	100.0	100.0	100.0	100.0	100.0
3pgh	34.5	43.9	50.4	57.4	64.4	42.7	25.4	30.3	51.6	25.8	95.7
3pgt	71.6	69.6	69.3	65.6	64.5	61.7	70.7	72.2	66.3	65.3	86.1
4cox	20.5	22.5	25.5	36.5	46.4	48.6	26.3	26.9	29.9	34.4	75.6
6cox	37.8	41.6	51.4	66.7	74.1	28.2	50.1	30.4	53.2	20.5	67.3
Average of AUC	65.8	67.4	67.9	69.4	68.6	44.6	68.2	67.0	68.2	64.5	74.6
of AUC	28.5	26.5	23.3	19.2	16.5	19.3	26.0	27.6	21.4	29.9	15.9
1% hit ratio	13.5	13.6	17.7	18.4	18.7	9.0	18.4	16.0	20.0	20.0	28.9

The λ [of equatoin (2)] dependence of the AUC value was examined with a change in λ from 0 to 1. The present combination of MVO with the docking method using λ = 0 is equivalent to the original Sievgene docking program, and that with λ = 1 is equivalent to the score of the original MD-MVO method. The optimal λ value was found to be 0.5 from Table 1 and Table 2. This trend did not depend on the difference in decoy sets. Considering the AUC values in Table 1 and Table 2, the optimal λ value should exist between 0.5 and 0.65. The trends of the 1% hit ratio and the AUC values are convex upward between λ =0 and λ =1, we selected the λ =0.5 that mean the simple average of the docking score and the MVO score for further consideration. The Sievgene docking scores were distributed from -2 to -4, and the MVO scores were distributed from 0 to -1. The hit ratio was almost proportional to the AUC value. Namely, the correlation coefficients between the hit ratio and the AUC were 0.53 and 0.75 for the LigandBOX decoy set and the Coelacanth decoy set, respectively. Thus, we used the AUC value in the following discussions.

The van der Waals radius dependence of the AUC value was also examined. The van der Waals radii of the protein atoms were reduced by the damping factor (*k*), which was changed from 1 to 0.8. The protein structure was fixed in the compound docking process, whereas in the docking process it should change as induced fitting. To mimic the induced fitting and reduce the atomic conflict between the compound and the protein, the van der Waals radius of protein was reduced by the damping factor. Although the AUC value depends strongly on the λ value, it does not strongly depend on the damping factor, the optimum value of which is 0.85 to 0.95.

The screening results obtained by the combined MVO with docking method were better than the results obtained by the original Sievgene scoring method and those obtained by the MD-MVO method. Thus, the combination of the Sievgene score and the MVO score worked well for both compound sets. Also, the deviation in the hit ratios (AUC values) obtained by the combined MVO with docking method was smaller than those obtained by the original Sievgene score and the MVO score. This suggests that the hit ratio by the combined MVO with docking method is stable for many target proteins. The in-silico docking screening results strongly depend on both the protein and compound data sets. Perfect shape and electrostatic complementarities between protein and compound show strong affinity. In this case, slight structure changes of the protein or the compound should cause the atom confliction and the repulsive interaction or very weak affinity should be observed. In many docking studies including the current study, the protein structure is rigid and the ligand flexibility is partially considered (only rotatable bonds are rotated and the bond length/angle are fixed). Recently, ensemble docking approach is applied to reduce this problem [25,26,27]. In the current method, the ligand-based score (MVO score) should contribute to reduce the structure-dependence of the screening results. 

The large value of σ in Table 2 comparing to that in Table 1 was due to the low AUC values for COX-2. The AUC values of COX-2 (1cox, 1pxx, 3pgh, 4cox and 6cox) were almost half of the average value of AUC. The decoy set of Table 2 was provided by only one company (Coelacanth Co. Ltd.) and the decoy set of Table 1 was provided by 45 companies. The compound provided by Coelacanth was a random library but it could be biased to bind COX-2. If the Coelacanth decoy set was less random than the LigandBOX decoy set, the σ values in Table 2 should be bigger than those in Table 1.

**Table 3:** AUC values (%) obtained by the combined MVO method for DUD decoy set.

Method	Combined MVO docking			MCS
λ	0	0.5	1	
4cox	63.2	63.4	47.0	63.6
6cox	51.6	53.8	42.9	73.8
3ert	73.3	72.0	63.4	91.2
3erd	57.4	57.7	52.3	94.1
1hpv	43.1	42.3	64.0	68.9
1htf	50.1	51.6	53.2	15.4
1etr	63.5	59.6	45.1	86.7
1ets	60.1	54.7	44.1	75.5
1tng	74.7	73.0	37.2	55.6
1tnh	75.5	68.5	36.4	58.0
Average	61.3	59.7	48.6	68.3
	10.5	9.2	9.2	21.7
1% hit ratio	6.3	4.2	0.6	10.0

We also applied the maximum common substructure search [28] with the ranking by the Tanimoto index as the similarity search method. The results were shown in Table 1 and Table 2. The screening results obtained by the combined MVO with docking method were slightly better than the results obtained by the obtained by the MCS method, when the LigandBOX decoy set and the both results were similar to each other when the Coelacanth decoy set was used.

The present method was applied to the drug screening of five target proteins with using the DUD: COX-2, ER, HIV, THR, and TRY [29]. Ten screening results were obtained. The AUC values of the 10 screening results are summarized in Table 3. The screening results obtained by the combined MVO with docking method were slightly worse than the results obtained by the original Sievgene scoring method and those obtained by the MCS method. But the deviation of the AUC value obtained by the combined MVO with docking method were smaller than the results obtained by the original Sievgene scoring method and those obtained by the MCS method. The method with small AUC deviation is useful, since the hit ratio does not vary widely for each target. The screening results obtained by the combined MVO with docking method were different from the results obtained by the MCS method. Namely, the correlation coefficient between the ranking orders by the combined MVO with docking and the MSC methods was only 0.38. The users can use both the methods and try the consensus results of them.

The MS-MTS method does not require protein-ligand complex structures. On the contrary, the combined MVO with docking method requires at least a single protein-ligand complex structure, but the number of active compounds could be only one. Thus, the combined MVO with docking method should be suitable for fragment screening by X-ray crystallography in which one protein-ligand complex structure is provided. And the MS-MTS method should be suitable for a conventional HTS assay.

The Sievgene docking score consists of an accessible surface term (mainly hydrophobic interaction), an electrostatic term, a hydrogen bonding term, and a van der Waals term. The contributions of the accessible surface term, electrostatic term, hydrogen bonding term, and van der Waals term were 89.9%, 0.88%, 5.41%, and 3.78%, respectively. Usually, the hydrophobic interaction gives almost the same contribution as the hydrophilic interaction, and obviously the Sievgene docking score underestimates the hydrophilic interaction. The suitable charge distribution of the potentially active compound could not be predicted by the Sievgene docking score. On the other hand, the MVO method considers the charge distribution of the compound based on the known active compound, but the MVO score cannot evaluate the protein-compound interaction. Thus, the combination of the Sievgene docking score and the MVO score should compensate for the weak point of each scoring method.

We applied our method to the estrogen receptor alpha (ER-α) that is a transcription factor and has its agonists, antagonists and modulators. A complex of ER- α with estrogen (PDB ID: 1ere) was selected as the template and the ligands of the other complex structures were docked into ER- α pocket (Table 4). Estrogen is an agonist of ER- α, and most of the ligands are antagonists or modulators. Table 4 shows the RMSD values between the experimental and docked coordinates of ligand heavy atoms were calculated. The main-chain RMSDs of proteins were also calculated. The conventional docking (λ=0), the MVO method (λ=1) and the combined-MVO with docking method (λ=0.5) were examined and the current method slightly improved the docking accuracy. As shown in Table 4, the ligand structures were not steroid scaffold that is a template ligand structure. Since the docking poses of these compounds were improved by the combined-MVO with docking method, the current method could be applied to various structurally different molecules from the template molecule to improve the docking poses.

**Table 4:** Ligand-binding posesobtained by the docking methods for estrogen receptor-α (ER- α).

PDB ID	Scaffold of ligand	RMSD(Å) (protein)	RMSD (Å)(ligand)		
			λ=0	λ =0.5	λ =1
1ere	Estrogen (steroid)	0.00	6.90	6.60	6.22
1l2i	Tetahydrochrysene	0.40	2.43	0.65	3.49
3uuc	Bisphenol	0.56	5.23	4.65	1.12
3erd	Triphenylethylene	0.61	2.90	2.84	4.12
2iok	Indole	0.78	2.97	2.09	6.17
1err	Benzothiophen	0.79	6.21	6.15	9.85
1r5k	Triphenylethylene	0.79	7.00	6.50	7.60
1yin	Chromane	1.25	3.05	3.03	7.52
1sj0	Benzoxathin	1.29	7.52	7.61	6.74
2ouz	Tetrahydronaphthalen	1.30	8.48	8.39	5.92
1xp9	Benzoxathin	1.31	2.24	0.98	6.57
1xp6	Benzoxathin	1.32	2.84	3.61	9.42
1xpc	Benzoxathin	1.33	1.72	2.54	8.87
1xp1	Benzoxathin	1.34	2.45	2.64	9.16
1yim	Chromane	1.37	2.67	2.36	5.20
2iog	Indole	1.49	7.19	7.59	9.39
3ert	Triphenylethylene	1.57	2.68	2.62	8.15
Averaged RMSD (Å)			4.38	4.17	6.79

We examined the correlation between the experimental protein-ligand binding free energy and the calculated score. Thrombin (PDB IDs: 1dwb, 1etr, 1ets and 1ett) and trypsin (PDB IDs: 1ppc, 1pph, 1tng, 1tnh and 3ptb) were selected as targets. For thrombin, 1dwb was selected as a template and the other protein-ligand complex structures were superimposed onto the coordinates of 1dwb. For, trypsin, 1ppc was selected as a template and the other protein-ligand complex structures were superimposed onto the coordinates of 1ppc. The effect of the induced fitting was small. Namely, the main-chain RMSDs of thrombin and trypsin were less than 0.7 Å and 0.3 Å, respectively. The conventional docking (λ=0), the MVO method (λ=1) and the combined-MVO with docking method (λ=0.5) were examined and the current method did not improved the correlation between the binding free energy and the score. For thrombin, the correlation coefficients were 0.94, 0.56 and 0.29 for Sievgene docking score, the S_combined-MVO_ score and the MVO score, respectively. For trypsin, the correlation coefficients were 0.92, 0.90 and 0.40 for Sievgene docking score, the S_combined-MVO_ score and the MVO score, respectively. In practical use, we should try to find ligands with stronger affinity than the template ligand that is an initial weak hit compound. Thus, the similarity between ligands should not correlate with the binding affinity.

The combined MVO with docking method could be applied to drug design. When a desirable compound is designed, a set of compounds that are similar to the designed compound could be found by this method, considering the protein-compound interaction. When a designed compound cannot be synthesized, we can get similar compounds as an alternative from the commercially available compound library.

## 3. Methods: Combining MVO with the Docking Method

Protein-compound docking simulation was performed by the program, Sievgene, which is a protein-ligand flexible docking program for *in silico* drug screening [13]. This program generates many conformers (100 conformers by default) for each compound and keeps the target protein structure rigid, but with soft interaction forces adapting its slight structural change to some extent. There have been more than 100 protein-compound docking programs reported and the algorithms were summarized in a review [30]. There are mainly three types of scoring functions and these functions are based on inter-atomic distances among the protein and the compound atoms. Such scoring functions are strongly depending on the structure change of the protein, thus ensemble docking or consensus scoring has been used to overcome the problem. Sievgene scoring function was designed to consider the structural change of the target protein. In the inner region of the target protein, the protein is approximated as an elastic body, while the atomic pair-wise scoring function is applied in the outer region of the target protein. This docking program was developed with a performance yielding about 50% of the reconstructed complexes at a distance of less than 2 Å RMSD for the 132 complexed receptors with the compounds in PDB. The results predicted by our program were almost the same as those predicted by other docking programs [13]. The docking score (*H_dock_*) to estimate the protein-ligand binding free energy was determined as

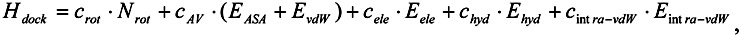
(8)
where *N_rot_, E_ASA_, E_vdW_, E_ele_, E_hyd_*, and *E_intra-vdW_* represent the number of rotatable bonds of the ligand molecule, the hydrophobic energy due to the accessible surface area, the vdW energy, the protein-ligand Coulombic potential, the hydrogen bond energy, and the intramolecular vdW energy of the ligand for Sievgene [13]. Also, *c_rot_, c_AV_, c_ele_, c_hyd_*, and *c_intra-vdW_* are the optimized coefficient for each energy term. For each atom type, the sum of *E_ASA_* and *E_vdW_* gives a grid potential, and both energy terms are always simultaneously calculated. Thus, these two terms share the same coefficient, *c_AV_*. Sievgene utilizes the grid potential to calculate each energy term except for the intramolecular interactions. In this study, a mesh size of 60 × 60 × 60 was adopted. The docking procedure was as follows (see Figure 1):

Step 1 The pocket is indicated by the known ligand coordinates, and the potential energy grids were generated around the ligand-binding pocket.
Step 2 Electrostatic potential field on the accessible surface of the receptor is calculated to find a total of 30 potential minima and maxima. Also, hydrophobic potential is calculated by using a methane probe to find those 30 potential minima. Triangles are generated to connect these points; the data regarding these triangles are recorded in a hash table.
Step 3 The program reads a compound of the database and then generates its conformers. The dihedral angles are randomly incremented every 120 degrees.
Step 4 The global search program chooses any three atoms of the compound and superimposes the compound onto the receptor surface according to the geometric hash method. The *S_combined-MVO_* score is then evaluated.
Step 5 Starting from the initial coordinate generated in step 4, the compound coordinates reaches the optimal complex structure using the steepest descent method to minimize the *S_combined-MVO_* score with the grid potential of the receptor force field and the known-ligand coordinates. The AMBER-type molecular force field is used.

In the present study, the docking simulations were performed by a modified version of the Sievgene/myPresto program.

## 4. Preparation of Materials

To evaluate our method, we performed a protein-compound docking simulation based on the soluble protein structures registered in the Protein Data Bank (PDB). Our target proteins and the inhibitors were almost equal to those used in our previous work [31]. The target proteins were the macrophage migration inhibitory factor (PDB code: 1gcz), COX-2 (PDB code: 1cx2, 1pxx, 3pgh, 4cox and 6cox), HIV protease-1 (PDB code: 1aid, 1hpx and 1ivp), thermolysin (PDB code: 1tmn, 2tmn, 1tlp), and GST (PDB code: 18gs, 2gss, and 3pgt), and carboxypeptidase A (PDB code: 1cbx, 1cps, 3cpa). The protein-ligand complex structures were selected from the PDB, and the hydrogen atoms were added for the present docking study. The atomic charges of the proteins were the same as the atomic charges of AMBER parm99 [32].

The compound set consisted of 14 inhibitors of MIF, 28 inhibitors of thermolysin, 14 inhibitors of COX-2, 19 inhibitors of HIV protease-1, 12 inhibitors of GST, and 3 inhibitors of carboxypeptidase A [31]. Two decoy sets were prepared. One is a set of 11,050 potential-negative compounds of the Coelacanth chemical compound library (Coelacanth Corporation, East Windsor, NJ, USA) which is a random library, and the other is a set of 10,000 randomly selected compounds from the LiganBOX database [24]. The LigandBOX data were provided by 45 companies, while the Coelacanth decoy set was provided by only one company. The Coelacanth decoy was bigger than the LigandBOX decoy. The average mass weight of the Coelacanth decoy set was 423Da and the average number of heavy atoms was 30.9. The mass weight of each compound of the LiganBOX decoy set was > 150 Da and < 350 Da, the average mass weight was 289 Da and the average number of heavy atoms was 20.4. The molecular size of the DUD decoy set was intermediate and the size depended on the target protein. The average mass weight was 300-450 Da and the average number of heavy atoms was 20-35.

The 3D coordinates of the Coelacanth chemical compound library were generated by the Concord program (Tripos, St. Louis, MO, USA) from the 2D Sybyl SD files provided by the Coelacanth Chemical Corporation. The 3D coordinates of the inhibitors were generated by Chem3D (Cambridge Software, Cambridge, MA, USA). The atomic charges of each ligand were determined by the Gasteiger method [33,34]. The LigandBOX library generation procedure was described in our previous paper [24].

The other compound set was the decoy set of the directory of useful decoy (DUD) for each target protein [29]. The specific DUD decoy and ligand sets were prepared for each target. The cyclooxygenase-2 (COX2), estrogen receptor (ER), HIV protease-1 (HIV), thrombin (THR), and trypsin (TRY) were selected for the validation test of the current method. Two target protein structures were selected for each of the other five proteins. Namely 4cox and 6cox for COX2, 3erd and 3ert for ER, 1hpv and 1htf for HIV, 1etr and 1ets for THR, and 1tng and 1tnh for TRY were used, respectively. The compound set consisted of inhibitors of a target protein and compounds of a decoy set. The numbers of prepared inhibitors or antagonists were 426, 39, 62, 72, and 49 for COX2, ER, HIV, THR, and TRY, respectively. The numbers of compounds of DUD decoy sets for COX2, ER, HIV, THR and TRY were 13289, 1448, 2038, 2456, and 1664, respectively.

## 5. Conclusions

We have developed a combined MVO with docking method, which is an integration of SBDS and LBDS. This method can be applied when at least a single protein-ligand complex structure is available. Compounds from a database were docked around the known-ligand coordinates by a flexible docking method. The volume overlap between the known ligand and the docked compound was calculated, and the total score was the sum of the volume overlap and the protein-compound binding energy. The combined MVO with the docking method was applied to *in silico* drug screenings for several target proteins. The hit ratio obtained by this combined method was higher than that achieved in a native docking-screening study, and it was better than the results obtained by the original Sievgene docking score and the MVO score. 

## References

[1] van de Waterbeemd H., Testa B., Folkers G. (1997). Computer-Assisted Lead Finding and Optimization –Current Tools for Medicinal Chemistry.

[2] Leach A.R. (2001). Molecular Modeling–Principles and Applications.

[3] Richards W.G., Robinson D.D., Truhlar D.G., Howe W.J., Hopfinger A.J., Blaney J., Dammkoehler R.A. (1999). Rational Drug Design.

[4] Pickett S., Boehm H.J., Schneider G., Mannhold R., Kubinyi H., Folkers G. (2003). Protein-Ligand Interactions from Molecular Recognition to Drug Design–Methods and Principles in Medicinal Chemistry.

[5] Pearlman R.S., Smith K.M. (1999). Metric validation and the receptor-relevant subspace concept. J. Chem. Inf. Compt. Sci..

[6] Fukunishi Y., Nakamura H. (2008). Prediction of protein-ligand complex by docking software guided by other complex structures. J. Mol. Graph. Model..

[7] Fukunishi Y., Nakamura H. (2009). A new method for *in silico* drug screening and similarity search using molecular dynamics maximum volume overlap (MD-MVO) method. J. Mol. Graphics Mod..

[8] Kuntz I.D., Blaney J.M., Oatley S.J., Langridge R., Ferrin T.E. (1982). A geometric approach to macromolecule-ligand interactions. J. Mol. Biol..

[9] Rarey M., Kramer B., Lengauer T., Klebe G. (1996). A fast flexible docking method using an incremental construction algorithm. J. Mol. Biol..

[10] Jones G., Willet P., Glen R.C., Leach A.R., Taylor R. (1997). Development and validation of a genetic algorithm for flexible docking. J. Mol. Biol..

[11] Goodsell D.S., Olson A.J. (1990). Automated docking of substrates to proteins by simulated annealing. Proteins.

[12] Abagyan R., Totrov M., Kuznetsov D. (1994). ICM: a new method for structure modeling and design: application to docking and structure prediction from the disordered native conformation. J. Compt. Chem..

[13] Fukunishi Y., Mikami Y., Nakamura H. (2005). Similarities among receptor pockets and among compounds: Analysis and application to *in silico* ligand screening. J. Mol. Graphics Mod..

[14] Neves M.A.C., Totrov M., Abagyan R. (2012). Docking and scoring with ICM: the benchmarking results and strategies for improvement. J. Comput. Aided. Mol. Des..

[15] Spitzer R., Jain A.N. (2012). Surflex-Dock: docking benchmarks and real-world application. J. Comput. Aided. Mol. Des..

[16] Schneider N., Hindle S., Lange G., Klein R., Albrecht J. (2012). Substantial improvements in large-scale redocking and screening using the novel HYDE scoring function. J. Comput. Aided Mol. Des..

[17] Novikow F.N., Stroylov V.S., Zeifman A.A., Stroganov O.V., Kulkov V. (2012). Lead Finder docking and virtual screening evaluation with Astex and DUD test sets. J. Comput. Aided Mol. Des..

[18] Liebeschuetz J.W., Cole J.C., orb O. (2012). Pose prediction and virtual screening performance of GOLD scoring functions in a standardized test. J. Comput. Aided Mol. Des..

[19] Brozell S.R., Mukherjee S., Balius T.E., Roe D.R., Case D.A. (2012). Evaluation of DOCK 6 as a pose generation and database enrichment tool. J. Comput. Aided Mol. Des..

[20] Corbeil C.R., Williams C.I., Labute P. (2012). Variability in docking success rates due to dataset preparation. J. Comput. Aided Mol. Des..

[21] Repasky M.P., Murphy R.B., Banks J.L., Greenwood J.R., Tubert-brohman I.  (2012). Docking performance of the glide program as evaluated on the Astex and DUD datasets: a complete set of glide SP results and selected results for a new scoring function integrating WaterMap and glide. J. Comput. Aided Mol. Des..

[22] Christofferson A.J., Huang N. (2012). Computational Drug Discovery and Design.

[23] Fukunishi Y., Kubota S., Nakamura H. (2006). Noise reduction method for molecular interaction energy: application to in silico drug screening and in silico target protein screening. J. Chem. Info. Mod..

[24] Fukunishi Y., Sugihara Y., Mikami Y., Sakai K., Kusudo H., Nakamura H. (2009). Advanced in-silico drug screeing to achieve high hit ratio−development of 3D-compound database. Synthesiology.

[25] Cosconati S., Marinelli L., Leva F.S.D., Pietra V.L., Simone A.D., Mancini F., Andrisano V., Novellino E., Goodsell D.S., Olson A.J. (2012). Protein flexibility in virtual screening: the BACE-1 case study. J. Chem. Inf. Model..

[26] Rueda M., Totrov M., Abagyan R. (2012). ALiBERO: evolving a team of complementary pocket conformations rather than a single leader. J. Chem. Inf. Model..

[27] Wada M., Kanamori E., Nakamura H., Fukunishi Y. (2011). Selection of in-silico drug screening results for G-protein-coupled receptors by using universal active probe. J. Chem. Inf. Model..

[28] Kawabata T. (2011). Build-up algorithm for atomic correspondence between chemical structures. J. Chem. Info. Mod..

[29] Huang N., Shoichet B.K., Irwin J.J. (2006). Benchmarking sets for molecular docking. J. Med. Chem..

[30] Huang S.Y., Zou X. (2010). Advances and challenges in protein-ligand docking. Int. J. Mol. Sci..

[31] Fukunishi Y., Nakamura H. (2008). Improvement of protein-compound docking scores by using amino-acid sequence similarities of proteins. J. Chem. Info. Mod..

[32] Case D.A., Darden T.A., Cheatham T.E.III., Simmerling C.L., Wang J., Duke R.E., Luo R., Merz K.M., Wang B., Pearlman D.A., Crowley M., Brozell S., Tsui V., Gohlke H., Mongan J., Hornak V., Cui G., Beroza P., Schafmeister C., Caldwell J.W., Ross W.S., Kollman P.A. (2004). AMBER 8.

[33] Gasteiger J., Marsili M. (1980). Iterative partial equalization of orbital electronegativity—A rapid access to atomic charges. Tetrahedron.

[34] Gasteiger J., Marsili M. (1978). A new model for calculating atomic charges in molecules. Tetrahedron Lett..

